# Phylogeny and taxonomy of *Lycoperdaceae*: *Longipediperdon* gen. nov., a new genus from China based on molecular and morphological evidence

**DOI:** 10.3897/mycokeys.136.188385

**Published:** 2026-07-15

**Authors:** Xin Yang, Songjing Duan, Simiao Li, Xin Gao, Run Yang, Dede Jiang, Hongmin Zhou, Changlin Zhao, Shan Shen

**Affiliations:** 1 The Key Laboratory of Forest Resources Conservation and Utilization in the Southwest Mountains of China Ministry of Education, Yunnan Provincial Key Laboratory for Conservation and Utilization of In-forest Resource, Southwest Forestry University, Kunming 650224, China Guangxi Zhuang Autonomous Region State-owned Qipo Forest Farm Nanning China; 2 College of Forestry, Southwest Forestry University, Kunming 650224, China Department Microbial Drugs (MWIS), Helmholtz-Centre for Infection Research Braunschweig Germany; 3 Guangxi Zhuang Autonomous Region State-owned Qipo Forest Farm, Nanning 530225, China College of Forestry, Southwest Forestry University Kunming China; 4 Department Microbial Drugs (MWIS), Helmholtz-Centre for Infection Research, Braunschweig 38124, Germany The Key Laboratory of Forest Resources Conservation and Utilization in the Southwest Mountains of China Ministry of Education, Yunnan Provincial Key Laboratory for Conservation and Utilization of In-forest Resource, Southwest Forestry University Kunming China; 5 Modern Industry School of Edible-fungi, Southwest Forestry University, Kunming 650224, China Modern Industry School of Edible-fungi, Southwest Forestry University Kunming China; 6 College of Biological Science and Food Engineering, Southwest Forestry University, Kunming 650224, China College of Biological Science and Food Engineering, Southwest Forestry University Kunming China

**Keywords:** Classification, macrofungi, molecular systematics, new taxa, puffballs

## Abstract

Members of the family *Lycoperdaceae*, commonly known as puffballs, are characterized by their distinct gasteroid basidiomata. In this study, a new genus, *Longipediperdon***gen. nov**., and a new species, *Longipediperdon spineum***sp. nov**., from southern China are proposed based on morphological characteristics and molecular evidence. Morphologically, *Longipediperdon spineum* is distinguished by an exoperidium with densely arranged spines and basidiospores with remarkably long pedicels (4–15.5 µm). Multilocus phylogenetic analyses based on ITS, nLSU, and *rpb2* sequences support the establishment of these taxa within *Lycoperdaceae*. Specifically, *Longipediperdon spineum* forms an independent monophyletic lineage. Full morphological descriptions and illustrations are provided.

## Introduction

Fungi, encompassing mushrooms, molds, lichens, yeasts, and zoosporic forms, are ubiquitous heterotrophic organisms present across all terrestrial and aquatic ecosystems (Hibbett et al. 2025). As one of the most evolutionarily successful lineages, fungi exhibit remarkable morphological diversity and fulfill essential ecological roles as symbionts, parasites, and saprobes (Merényi et al. 2023). They play a central role in nutrient cycling by driving the decomposition of organic matter and mediating the flux of carbon, nitrogen, and phosphorus (Tedersoo et al. 2014; Treseder and Lennon 2015). Despite their ecological significance, global fungal diversity remains poorly documented. Only approximately 155,000 species have been formally described out of an estimated 2.5 million worldwide (Hibbett et al. 2025).

Representing a substantial portion of this unexplored diversity, the class *Agaricomycetes (Basidiomycota)* is highly diverse and occupies nearly all terrestrial habitats, ranging from tropical rainforests to Arctic tundra (Lutzoni et al. 2018; Varga et al. 2019). This class comprises mushrooms, bracket fungi, and gasteroid fungi, including the family *Lycoperdaceae* (commonly known as puffballs), which is characterized by gasteroid basidiomata that are globose, subglobose, depressed globose, pyriform, or obpyriform (Pegler et al. 1995; Li et al. 2025). Members of this family contribute significantly to organic matter decomposition and the bioaccumulation of heavy metals across temperate, arid, and tropical regions (Petkovšek and Pokorny 2013). Beyond their ecological functions, many *Lycoperdaceae* species possess notable economic and medicinal value; for instance, certain *Calvatia* species are utilized as food sources and in traditional medicine for their hemostatic properties (Van Wyk and Coetzee 2009; Li et al. 2024).

The family *Lycoperdaceae* was established in 1820 with *Lycoperdon* Pers. as the type genus (Berchtold and Presl 1820). Historically, it comprised approximately 18 genera and 150 species and was initially treated as synonymous with *Agaricaceae* (Kirk et al. 2008). Subsequent molecular phylogenetic analyses demonstrated that *Lycoperdaceae* represented a monophyletic gasteroid lineage distinct from *Agaricaceae* sensu stricto (He et al. 2024). Later studies identified four principal subclades within the family, namely *Bovista* Pers., *Calvatia* Fr., *Disciseda* Czern., and *Lycoperdonsensu lato* (Larsson and Jeppson 2008). In recent taxonomic treatments of *Basidiomycota*, *Lycoperdaceae* is recognized as an independent family encompassing genera such as *Apioperdon* (Kreisel & D. Krüger) Vizzini, *Bovista*, *Bryoperdon* Vizzini, *Calvatia*, *Calbovista* Morse ex M.T. Seidl, *Gastropila* Homrich & J.E. Wright, *Lycoperdon*, and *Morganella* Zeller (He et al. 2024). Nevertheless, several puffball-like genera, including *Abstoma* G. Cunn., *Acutocapillitium* P. Ponce de León, *Arachnion* Schwein., *Calvatiopsis* Hollós, *Disciseda*, *Glyptoderma* R. Heim & Perr.-Bertr., *Japonogaster* Kobayasi, and *Lycoperdopsis* Henn., have been retained within *Agaricaceae* (He et al. 2024). Although Kalichman et al. (2020) proposed a broad concept of *Agaricaceae* sensu lato covering *Lycoperdaceae*, *Coprinaceae*, *Lepiotaceae*, and *Tulostomataceae*, *Lycoperdaceae* has largely been reaffirmed as a distinct family in other major phylogenetic studies (He et al. 2024). Although Index Fungorum and MycoBank (accessed 7 February 2026) record more than 1,400 specific and infraspecific names within *Lycoperdaceae*, the number of currently accepted species is estimated to be 430 (He et al. 2024; Li et al. 2025; Yang et al. 2025b).

Historically, generic delimitation within *Lycoperdaceae* relied primarily on morphological characters. Diagnostic features included the type of capillitium, e.g., *Lycoperdon*-type, *Bovista*-type, or *Calvatia*-type, the structure of the endoperidial apical opening, and the presence or absence of a pseudostipe (Kreisel 1969; Pegler et al. 1995; Larsson and Jeppson 2008). *Lycoperdon* serves as the type genus of the family. Based on early molecular evidence, Larsson and Jeppson (2008) initially advocated for a broad circumscription of *Lycoperdon*, incorporating traditional genera such as *Bovistella* Morgan, *Morganella*, and *Vascellum* F. Šmarda. However, later studies demonstrated that subdividing *Lycoperdon* into five subgenera failed to satisfactorily accommodate the full breadth of taxa (Bates et al. 2009; Gube and Piepenbring 2009; Jeppson and Larsson 2010; Alfredo et al. 2017).

The circumscription of genera within *Lycoperdaceae* has since undergone substantial revision through the integration of morphological and molecular data (Vizzini and Ercole 2017; Krakhmalnyi et al. 2023). Recently, multilocus phylogenies inferred from ITS, nLSU, *rpb2*, and *TEF1*-*α* datasets have provided a robust temporal framework (Li et al. 2024, 2025). Divergence time estimates ranging from approximately 26.7 to 75.5 million years ago support the recognition of 19 genera within the family (Li et al. 2024). In addition, two additional genera, *Lycoperdia* Xin Yang & C.L. Zhao and *Tortoperdon* Kun L. Yang, Jia Y. Lin & Zhu L. Yang, were described using integrative taxonomic and phylogenetic approaches (Yang et al. 2025a, 2025b). Currently, *Lycoperdaceae* comprises a total of 29 recognized genera (Li et al. 2024, 2025; Yang et al. 2025c, 2025d).

During recent field investigations of gasteroid fungi in China, many puffball specimens were collected. The aim of the present study was to explore previously unrecognized taxa within *Lycoperdaceae* and clarify their phylogenetic relationships. Based on detailed morphological observations and multilocus phylogenetic analyses of ITS, nLSU, and *rpb2* sequences, a new genus, *Longipediperdon*, and a new species, *Longipediperdon spineum*, are herein described.

## Materials and methods

### Sample collection and examination

**Figure 1.**
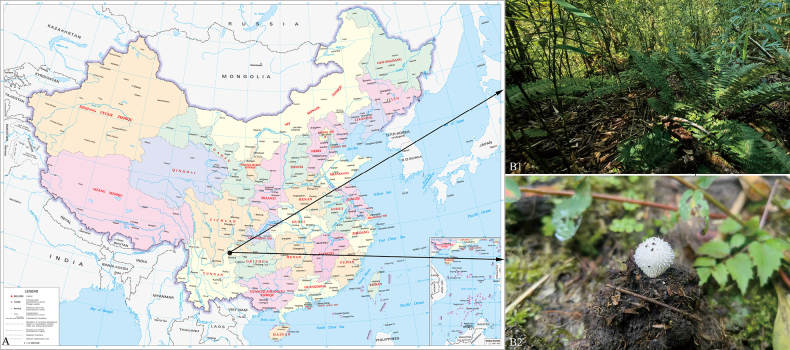
**A**. The collection localities of all specimens in this study. Map source: Standard map approved by the Ministry of Natural Resources of China, Approval No. GS (2024)4300; **B**. Morphological features of *Longipediperdon spineum* and its typical natural environment.

Fresh basidiomata growing on the ground were collected from Shaoyang, Hunan Province (Fig. 1), China. The specimens were photographed *in situ* using a digital camera (Xiaomi 12, China), and fresh macroscopic characteristics, including the color of the basidiomata, the type of exoperidium, and the shape of the basidiomata, were recorded. All photographs were focus-stacked and merged using Helicon Focus Pro 7.7.5 software. Specimens were dried in an electric food dehydrator at 40 °C (Dong et al. 2024; Yang et al. 2025b), then sealed in paper envelopes and deposited in the herbarium of Southwest Forestry University (**SWFC**), Kunming, Yunnan Province, P.R. China.

### Morphology

The macromorphological descriptions were based on field notes and photographs captured in the field and laboratory and followed the color terminology of Petersen (1996). Micromorphological data were obtained from the dried specimens following observation under a light microscope (Gu et al. 2025; Yang et al. 2025d). Drawings were made with the aid of a fungus plotter (Yang et al. 2025b). The ultrastructure of basidiospores was observed with scanning electron microscopy (SEM) using a field-emission scanning electron microscope (FESEM) GeminiSEM 360. The measurements and drawings were made from slide preparations stained with Cotton Blue (0.1 g aniline blue dissolved in 60 g pure lactic acid) and 5% potassium hydroxide. For spore size data, 5% of the measurements from each end of the range were excluded and shown in parentheses. The following abbreviations were used: KOH = 5% potassium hydroxide water solution, CB+ = cyanophilous, CB = cotton blue, CB– = acyanophilous, *Q* = variation in the *L*/*W* ratios between the specimens studied, and *n* = a/b (number of spores [a] measured from a given number [b] of specimens); *Qm* represents the average *Q* of basidiospores measured ± SD.

### DNA extraction, PCR amplification, sequencing, and phylogenetic analyses

The CTAB rapid fungi genome extraction kit-DN14 (Aidlab Biotechnologies Co., Ltd., Beijing, China) was used to obtain genomic DNA from the dried fungal specimens according to the manufacturer’s instructions (Dong et al. 2025a; Yang et al. 2025b, 2025c). The extracted DNA was maintained at –20 °C for long-term storage. Three molecular markers were investigated, i.e., internal transcribed spacer (ITS), nuclear large subunit ribosomal RNA (nLSU), and RNA polymerase II subunit 2 (*rpb2*); the primers and conditions are shown in Table 1. The PCR products were purified and sequenced at Kunming Tsingke Biological Technology Limited Company (Yunnan Province, China). All newly generated sequences were deposited in NCBI GenBank (https://www.ncbi.nlm.nih.gov/genbank/) (Table 2).

**Table 1.** Genes, primers, originating loci, PCR amplification procedures, and references used in this study.

**Nam**e	**Abbreviation**	**Name**	**Direction**	**Sequence (5'-3')**	**Annealing temperature (°C)**	**References**
Internal transcribed spacer region of the rDNA	ITS	ITS5	Forward	GGAAGTAAAAGTCGTAACAAGG	55	White et al. 1990
		ITS4	Reverse	TCCTCCGCTTATTGATATGC		
Nuclear large subunit ribosomal	nLSU	LR0R	Forward	ACCCGCTGAACTTAAGC	48	Vilgalys and Hester 1990
		LR7	Reverse	TACTACCACCAAGATCT		
RNA polymerase second largest subunit	*rpb*2	fRPB2-6F	Forward	TGGGGYATGGTNTGYCCYGC	60	Liu et al. 1999
		fRPB2-7cR	Reverse	CCCATRGCTTGYTTRCCCAT		

**Table 2.** List of species, specimens, and GenBank accession number of sequences used in this study. [New species is shown in bold; * type material].

**Species na**me	**Sample no**.	**Locality**	**GenBank accession no**.			**References**
			**ITS**	**nLSU**	***rpb*2**	
*Abstoma indicum*	UZ-04-19	India	MN231720	–	–	Altaf et al. 2022
*Abstoma purpureum*	KM162954	England	GQ981488	–	–	Unpublished
*Apioperdon pyriforme*	QL20170019	China	PP175742	PP175746	–	Li et al. 2024
*Apioperdon pyriforme*	ZRL20182005	China	PP175743	PP175747	–	Li et al. 2024
*Bovista cretacea*	ANMH11622	Iceland	DQ112611	DQ112611	–	Larsson and Jeppson 2008
*Bovista cretacea*	MJ5207	Norway	DQ112610	DQ112610	–	Larsson and Jeppson 2008
*Bovista khorchinensis*	FJAU71982*	China	PQ588042	PQ588044	–	Liu et al. 2025
*Bovista khorchinensis*	FJAU71983	China	PQ588043	PQ588045	–	Liu et al. 2025
*Bovista litangensis*	HMAS 258800*	China	OR792635	OR831301	OR853765	Li et al. 2024
*Bovista litangensis*	HMAS 258801	China	OR792636	OR831302	OR853764	Li et al. 2024
*Bovista nyalamensis*	HMAS 258836*	China	OR792637	OR831304	OR853767	Li et al. 2024
*Bovista nyalamensis*	HMAS 258837	China	OR792638	OR831306	OR853766	Li et al. 2024
*Bovista plumbea*	NYGD01	Pakistan	JX183694	–	–	Yousaf et al. 2013
*Bovista plumbea*	MJ4856	Sweden	DQ112613	DQ112613	–	Larsson and Jeppson 2008
*Bovistella emodensis*	HMAS 287485*	China	PP175744	PP175752	PP351955	Li et al. 2024
*Bovistella emodensis*	HMAS 287486	China	PP175745	PP175753	PP351956	Li et al. 2024
*Bovistella radicata*	parker970911	USA	DQ112608	DQ112608	–	Larsson and Jeppson 2008
*Bovistella utriformis*	MJ5388	Sweden	DQ112607	DQ112607	–	Larsson and Jeppson 2008
*Bryoperdon acuminatum*	TO HG191016 et	Italy	KY581201	KY581199	–	Vizzini and Ercole 2017
*Bryoperdon acuminatum*	TO HG201016	Italy	KY581202	KY581200	–	Vizzini and Ercole 2017
*Calvatia craniiformis*	Steinke001017	USA	DQ112625	–	–	Larsson and Jeppson 2008
*Calvatia gigantea*	MJ3566	Sweden	DQ112623	–	–	Larsson and Jeppson 2008
*Calvatia longisetulosa*	HMAS 258802*	China	OR792617	OR831229	OR853757	Li et al. 2024
*Calvatia longisetulosa*	HMAS 258803	China	OR792618	OR831230	OR853758	Li et al. 2024
*Calvatia nodulata*	BAFC 4549	Argentina	KY366490	–		Alfredo et al. 2014
*Calvatia phlebioides*	CL Zhao 33216	China	PV345681	PV345675	PV341017	Yang et al. 2025c
*Calvatia phlebioides*	CL Zhao 33366	China	PV345682	PV345676	PV341018	Yang et al. 2025c
*Calvatia* sp.	CLZhao 48826*	China	PX965488	PX971289	PX963978	Present study
*Calvatia* sp.	CLZhao 48965	China	PX965489	PX971290	–	Present study
*Calvatia shennongjiaensis*	HMAS 258804	China	OR792621	OR831294	OR853761	Li et al. 2024
*Calvatia shennongjiaensis*	HMAS 258806*	China	OR792623	OR831291	OR853759	Li et al. 2024
*Calvatia subbooniana*	HMAS 258809	China	OR792631	OR831296	–	Li et al. 2024
*Calvatia subbooniana*	HMAS 258810*	China	OR792630	OR831297	–	Li et al. 2024
*Disciseda bovista*	M. Jeppson 5078	Sweden	DQ112627	–	–	Larsson and Jeppson 2008
*Disciseda candida*	STB304	USA	EU833654	–	–	Bates et al. 2009
*Disciseda cervina*	FK17016	Argentina	MN338568	–	–	Unpublished
*Fuscospina nigrescens*	MJ5376	Sweden	DQ112577	–	–	Larsson and Jeppson 2008
*Fuscospina nigrescens*	HMAS 258881	China	OR792702	OR831256	PP351957	Li et al. 2024
*Fuscospina scabricapillitia*	HMAS 258812*	China	OR792678	PP175748	PP351958	Li et al. 2024
*Globaria aestivalis*	MJ1122	Sweden	DQ112620	–	–	Larsson and Jeppson 2008
*Globaria albata*	HMAS 258884*	China	OR792659	OR831270	OR853784	Li et al. 2024
*Globaria gyirongensis*	HMAS 258813*	China	OR792660	OR831288	OR853785	Li et al. 2024
*Globaria gyirongensis*	HMAS 258814	China	OR792661	OR831287	OR853786	Li et al. 2024
*Globaria himalaica*	NYGL5*	Pakistan	JX183690	–	–	Yousaf et al. 2013
*Globaria jingningensis*	HMAS 258815*	China	OR792644	OR831273	–	Li et al. 2024
*Globaria muscicola*	HMAS 258835*	China	OR792641	OR831268	OR853771	Li et al. 2024
*Globaria rufa*	ZRL20240903	China	PV484409	–	–	Li et al. 2025
*Globaria rufa*	ZRL20240406*	China	PV484408	–	–	Li et al. 2025
*Globaria shannanensis*	ZRL20152235*	China	OR792662	OR831303	–	Li et al. 2024
*Globaria testacea*	HMAS 258819*	China	OR792656	OR831280	OR853778	Li et al. 2024
*Holocotylon biconicum*	HMAS 258778*	China	OR792667	OR831266	–	Li et al. 2024
*Holocotylon brandegeeanum*	STB111*	USA	EU833660	–	–	Bates et al. 2009
*Holocotylon dermoxanthum*	MJ4568	Sweden	DQ112579	–	–	Larsson and Jeppson 2008
*Holocotylon purpurascens*	HMAS 258780*	China	OR792671	OR831300	OR853806	Li et al. 2024
*Holocotylon rupicola*	MJ4304	Norway	DQ112580	–	–	Jeppson et al. 2012
*Holocotylon rupicola*	M. Jeppson 7007	Sweden	JN572902	–	–	Jeppson et al. 2012
*Leptocaulis albiperidia*	KA12-1210	Korea	KP340182	–	KU764391	Kim et al. 2016
*Leptocaulis albiperidia*	KA12-1551 t	Korea	KP340183	–	–	Kim et al. 2016
*Leptocaulis ericaea*	MJ 5395	Sweden	DQ112605	–	–	Larsson and Jeppson 2008
*Leptocaulis ericaea*	KA13-1463	Korea	KP340185	–	KU764396	Kim et al. 2016
*Leptocaulis muscorum*	M. Jeppson 9017	Sweden	JN572905	–	–	Jeppson et al. 2012
*Leptocaulis sublongistipes*	HMAS 258775*	China	OR792741	OR831318	OR853825	Li et al. 2024
*Leptocaulis subumbrinus*	HMAS 258885	China	OR792673	OR831315	OR853828	Li et al. 2024
*Leptocaulis subumbrinus*	HMAS 258886	China	OR792675	OR831317	OR853830	Li et al. 2024
***Longipediperdon spineum***	HMZhou 1841*	**China**	**PX965490**	**PX971291**	**–**	**Present study**
***Longipediperdon spineum***	**HMZhou 1839**	**China**	**PX965491**	**PX971292**	**PX963979**	**Present study**
*Lycoperdia tomentosa*	CL Zhao 37502*	China	PV345683	PV345677	**PX963980**	Yang et al. 2025c; **present study**
*Lycoperdia tomentosa*	CL Zhao 45073	China	PV345684	PV345678	**PX963981**	Yang et al. 2025c; **present study**
*Lycoperdiscus lividus*	MJ4005	Sweden	DQ112600	–	–	Larsson and Jeppson 2008
*Lycoperdiscus lividus*	Dobremez 19740514	Nepal	DQ112599	–	–	Larsson and Jeppson 2008
*Lycoperdiscus tianzhuensis*	HMAS 258767	China	OR792726	–	–	Li et al. 2024
*Lycoperdiscus tianzhuensis*	HMAS 258766*	China	OR792725	OR831324	OR853822	Li et al. 2024
*Lycoperdon norvegicum*	MJ5453	Sweden	DQ112631	–	–	Larsson and Jeppson 2008
*Lycoperdon perlatum*	HMAS 258865	China	OR792758	OR831262	OR853791	Li et al. 2024
*Lycoperdon perlatum*	HMAS 258866	China	OR792763	OR831257	OR853792	Li et al. 2024
*Lycoperdon pseudoperlatum*	Liu 22	China	PP037938	–	–	Liu et al. 2024
*Lycoperdon pseudoperlatum*	Liu 193*	China	PP037939	–	–	Liu et al. 2024
*Lycoperdon* sp.	CLZhao 19186*	China	PX965492	PX971293	–	Present study
*Lycoperdon* sp.	CLZhao 48964	China	PX965493	PX971294	–	Present study
*Lycoperdon subperlatum*	HMAS 258873	China	OR792751	OR831261	OR853789	Li et al. 2024
*Lycoperdon subperlatum*	HMAS 258875	China	OR792750	OR831260	OR853790	Li et al. 2024
*Morganella fuliginea*	UFRN-Fungos 2582	Brazil	KU958339	KU958340	–	Alfredo et al. 2017
*Morganella fuliginea*	UFRN-Fungos 2586	Brazil	KU958343	KU958344	–	Alfredo et al. 2017
*Morganella minima*	CL Zhao 40537*	China	PV345685	PV345679	–	Yang et al. 2025c
*Morganella minima*	CL Zhao 45072	China	PV345686	PV345680	–	Yang et al. 2025c
*Morganella nuda*	UFRN-Fungos 2568	Brazil	KU958313	KU958314	–	Alfredo et al. 2017
*Morganella nuda*	UFRN-Fungos 1766	Brazil	KU958315	KU958316	–	Alfredo et al. 2017
*Morganella oblongata*	UFRN-Fungos 2570	Brazil	KU958355	KU958356	–	Alfredo et al. 2017
*Morganella tricolor*	HMAS 287487*	China	PP175741	PP175750	–	Li et al. 2024
*Mycenastrum corium*	MJ5467	Sweden	DQ112628	–	–	Larsson and Jeppson 2008
*Mycenastrum corium*	STB113	USA	EU833666	–	–	Bates et al. 2009
*Pseudoperdon subcretaceum*	M. Jeppson 9032	Sweden	JN572908	–	–	Jeppson et al. 2012
*Pseudoperdon medogense*	HMAS 258785	China	OR792746	OR831255	OR853824	Li et al. 2024
*Pseudoperdon medogense*	HMAS 258786*	China	OR792743	–	–	Li et al. 2024
*Sinoperdon caudatum*	GC920818	Sweden	DQ112633	–	–	Jeppson and Larsson 2010
*Sinoperdon gyirongense*	HMAS 258787*	China	OR792686	OR831248	OR853801	Li et al. 2024
*Sinoperdon gyirongense*	HMAS 258788	China	OR792699	OR831243	OR853799	Li et al. 2024
*Tortoperdon suspectum*	HKAS150757 (HT)	China	PX308896	PX309010	–	Yang et al. 2025b
*Tortoperdon suspectum*	HTBM1229	China	PX308897	PX309011	–	Yang et al. 2025b
*Utraria excipuliformis*	HMAS 258850	China	OR792705	OR831326	–	Li et al. 2024
*Utraria excipuliformis*	HMAS 258851	China	OR792703	OR831328	–	Li et al. 2024
*Vascellum curtisii*	HMAS 258878	China	OR792665	OR831258	OR853787	Li et al. 2024
*Vascellum curtisii*	HMAS 258879	China	OR792666	OR831259	OR853788	Li et al. 2024
*Vascellum intermedium*	STB091	USA	EU833667	–	–	Bates et al. 2009
*Vascellum pratense*	MJ5858	Czechia	DQ112556	–	–	Larsson and Jeppson 2008
*Vascellum pratense*	HMAS 258880	China	OR792664	PP175749	PP351953	Li et al. 2024

**Figure 2.**
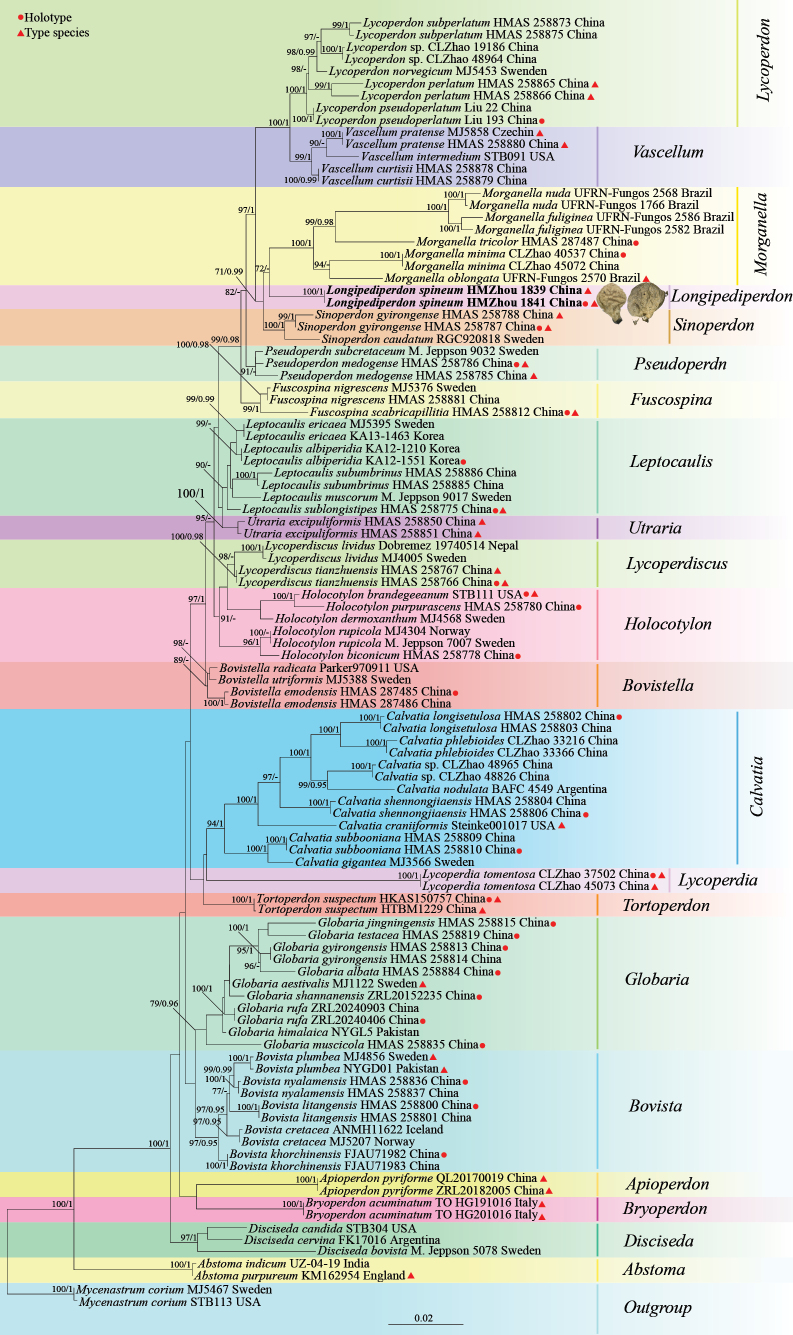
Maximum likelihood tree illustrating the phylogeny of *Longipediperdon* and related species in *Lycoperdaceae* based on ITS+nLSU+*rpb2* sequences. Branches are labeled with maximum likelihood bootstrap values ≥70% and Bayesian posterior probabilities ≥0.95, respectively. The new species is shown in bold.

Sequences generated for this study and additional sequences downloaded from GenBank were aligned. Sequences were aligned in MAFFT 7 (https://mafft.cbrc.jp/alignment/server/) by adjusting the direction of nucleotide sequences according to the first sequence (accurate enough for most cases) and selecting the G-INS-i iterative refinement method (Katoh et al. 2019). The alignment was adjusted manually using AliView version 1.27 (Larsson 2014). The dataset was aligned first, and then the sequences of ITS+nLSU+*rpb2* were combined with Mesquite v. 3.51. The combined ITS+nLSU+*rpb2* sequences and ITS+nLSU datasets were used to infer the position of the new species and related species. Sequences of *Mycenastrum corium* (Guers.) Desv. were retrieved from GenBank and used as outgroup taxa in the ITS+nLSU+*rpb2* analysis (Fig. 2) in the family *Lycoperdaceae* (Li et al. 2024).

Maximum likelihood (ML) and Bayesian inference (BI) analyses were applied to the combined two datasets following a previous study (Dong et al. 2025a). Phylogenetic reconstructions were performed using ML tools implemented in the PhyloSuite v1.2.3 platform (Zhang et al. 2020; Xiang et al. 2023). ML phylogenies were inferred with IQ-TREE v2.2.0, with 5,000 ultrafast bootstrap replicates and 1,000 SH-aLRT tests to assess branch support (Nguyen et al. 2015).

The best evolutionary model of each alignment was estimated using jModelTest (Guindon and Gascuel 2003; Posada 2008) under the Akaike information criterion. MrModeltest 2.3 (Nylander 2004) was used to determine the best-fit evolutionary model for the dataset for BI. BI was conducted through the Cipres Science Gateway (https://www.phylo.org/portal2/login!input.action) with two independent runs, performing 0.5 million replicates each for the dataset and sampling one tree every 1,000 generations. The first 25% of the sampled trees were discarded as burn-in, and the remaining trees were used to reconstruct a majority-rule consensus and calculate Bayesian posterior probabilities (BPP) of the clades (Miller et al. 2012).

## Results

### Phylogenetic analyses

The combined ITS+nLSU+*rpb2* dataset (Fig. 2) included sequences from 271 fungal specimens representing 69 species. The RAxML analysis of the combined dataset yielded a best-scoring tree (Fig. 2), with a final ML optimization likelihood value of –12506.513. The alignment contained a total of 2,455 bp. The matrix had 595 distinct alignment patterns. Estimated base frequencies were A = 0.255, C = 0.203, G = 0.277, and T = 0.264; substitution rates were AC = 1.59676, AG = 4.74103, AT = 2.12468, CG = 0.87811, CT = 8.45823, and GT = 1.000000. The best-fit BI model for the ITS dataset was GTR+I+G; the average standard deviation of split frequencies in the Bayesian analyses reached 0.007999, and the effective sample size (ESS) for Bayesian analysis across the two runs was double the average ESS (avg. ESS = 904). Branches that received ML bootstrap support ≥ 70% and BI posterior probabilities ≥ 0.95 were considered significantly supported.

The topology based on ITS+nLSU+*rpb2* sequences (Fig. 2) showed that the new genus and species nested within the family *Lycoperdaceae*, with *Longipediperdon spineum* forming an independent monophyletic lineage.

### Taxonomy

#### Longipediperdon

Taxon classificationFungiAgaricalesLycoperdaceae

Xin Yang & C.L. Zhao gen. nov.

AEF304BD-7680-520C-BEF3-BEEC2C12E9CF

MycoBank No: 862264

##### Type species.

*Longipediperdon spineum* Xin Yang & C.L. Zhao.

##### Etymology.

*Longipediperdon* (Lat.): referring to the basidiospores having a long pedicel.

##### Description.

Basidiomata subglobose to obpyriform. Exoperidium with densely arranged spiny structure. Endoperidium papery and membranous. Gleba cottony when young, pulverulent when mature. Exoperidium made up of chains of inflated cells, ellipsoid, oblong, thin-walled to slightly thick-walled, smooth, colorless hyphae in 5% KOH. Endoperidium made up of colorless hyphae, thick-walled, occasionally branched, without septa. Capillitium of *Lycoperdon*–type, occasionally branched, thick-walled, without septa. Paracapillitium absent. Basidiospores ovoid, and oblong, with short spines, with a long pedicel. Basidia bubble-shaped to subcylindrical and occasionally clavate, four sterigmata and a simple septum at the base.

#### Longipediperdon spineum

Taxon classificationFungiAgaricalesLycoperdaceae

Xin Yang & C.L. Zhao sp. nov.

EECA7880-D46C-5E40-BAE4-F47EAAB5E72C

MycoBank No: 862265

Figs 3, 4

##### Holotype.

China. • Yunnan Province, Zhaotong, Daguan County, Tianxing Town, Wumengshan National Nature Reserve, 27°52'50.4"N, 104°4'42.9"E, elev. 1882 m, on the ground in forest of *Chimonobambusa tumidissinoda*, 18 July 2025, HMZhou 1841 (SWFC 00101841).

**Figure 3.**
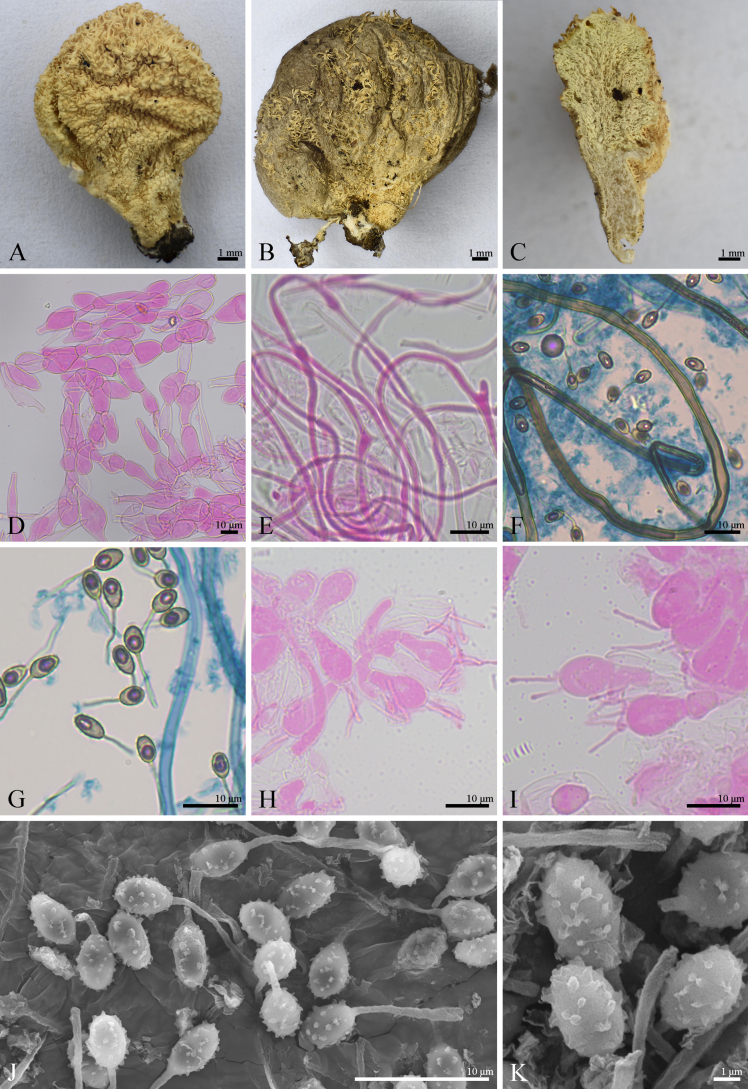
*Longipediperdon spineum* (HMZhou 1841). **A–C**. Basidiomata of *Lp. spineum*; **D**. Exoperidial elements; **E**. Endoperidial hyphae; **F**. Capliitium; **G**. Basidiospores; **H, I**. Basidia; **J, K**. Basidiospores under SEM.

**Figure 4.**
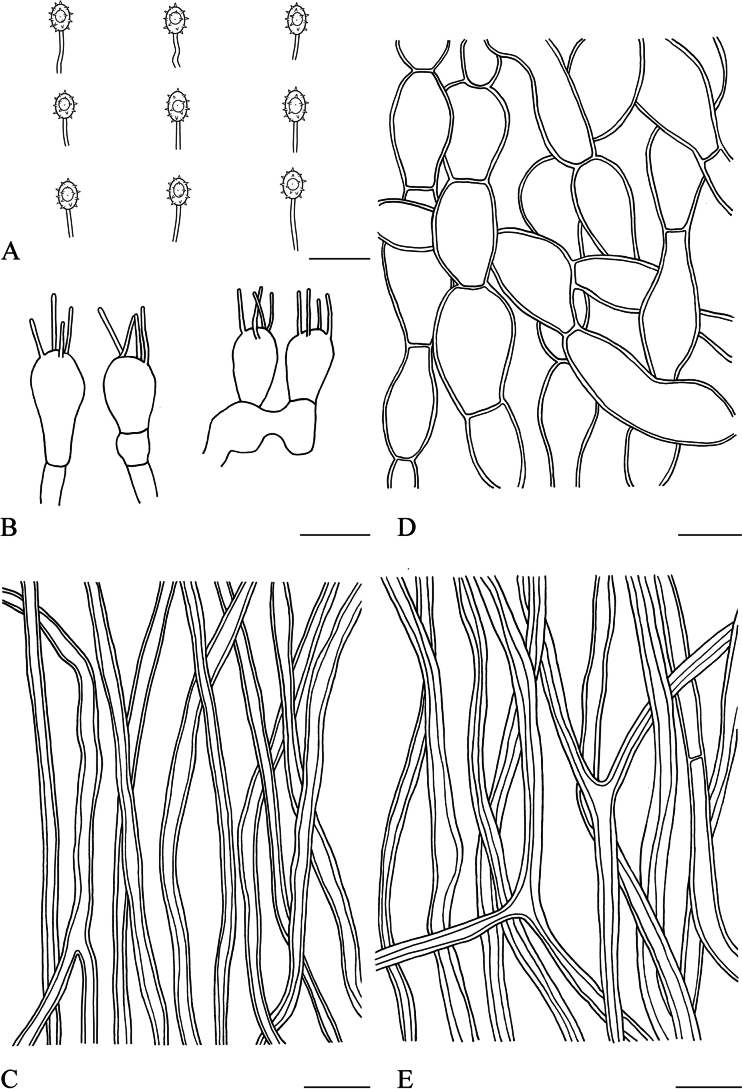
*Longipediperdon spineum* (HMZhou 1841). **A**. Basidiospores; **B**. Basidia; **C**. Capliitium; **D**. Exoperidial elements; **E**. Endoperidial hyphae. Scale bars: 10 µm (**A–E**).

##### Etymology.

*spineum* (Lat.): refers to the spines covering the surface of the basidiomata.

##### Fruiting body.

Basidiomata subglobose to obpyriform, 8–15 mm in diameter, 9–14 mm in height. Peridium layered: Exoperidium with densely arranged spiny structure, white when fresh, cream to pinkish-buff when dry, not easily fall off. Endoperidium papery to membranous, white when fresh, orangeyellow to ash-gray when dry. Gleba white when young, mouse-gray when mature, cottony when young, pulverulent when mature.

##### Hyphal structure.

Exoperidium composed of chains of inflated cells, ellipsoid to oblong, thin-walled to slightly thick-walled, colorless in 5% KOH, 9.5–31(–40) × (8–)9–13(–16.5) µm. Endoperidium made up of colorless hyphae, thick-walled, occasionally branched, without septa, 2.5–4(–5) μm in diameter. Capillitium of *Lycoperdon*-type, occasionally branched, thick-walled, without septa, 2.2–3.6(–4) μm in diameter. Paracapillitium absent.

##### Basidiospores.

Ovoid to oblong, colorless, thick-walled, CB–, (4.0–)4.4–5.8(–6.0) × (2.5–)2.9–3.2(–3.5) μm, *Q* = 1.58–1.65, *Qm* = 1.62 ± 0.16, spines (less than 1 µm), with a long pedicel (4–15.5 μm). Basidia bubble-shaped to subcylindrical, and occasionally clavate, four sterigmata and a simple septum at the base, 11–18 × (5.5–)7–8 µm.

##### Additional specimen examined

**(paratype)**. China. • Yunnan Province, Zhaotong, Daguan County, Tianxing Town, Wumengshan National Nature Reserve, 27°52'50.4"N, 104°4'42.9"E, elev. 1882 m, on the ground in forest of *Chimonobambusa tumidissinoda*, 18 July 2025, HMZhou 1839 (SWFC 00101839).

## Discussion

In the family *Lycoperdaceae*, several morphological characteristics serve as distinguishing features for its genera, including the type of capillitium, the ornamentation of basidiospores, the presence or absence of paracapillitium, the structure of the subgleba, the mode of dehiscence of the endoperidium, and the peridial anatomy (Larsson and Jeppson 2008; Jeppson and Larsson 2010; Krakhmalnyi et al. 2023; Li et al. 2024, 2025; Yang et al. 2025a, 2025b). In the present study, a new genus, *Longipediperdon*, and a new species, *Longipediperdon spineum*, are described based on phylogenetic analyses and morphological characteristics.

Phylogenetically, the phylogram based on the combined ITS+nLSU+*rpb2* sequences (Fig. 2) showed that the new genus *Longipediperdon* formed a monophyletic lineage, and the new species *Lp. spineum* was assigned to the genus *Longipediperdon* within the family *Lycoperdaceae*. However, morphologically, *Morganella* differs from *Longipediperdon* in its absent capillitium (Li et al. 2024).

Morphologically, *Longipediperdon* resembles 17 other genera in the family *Lycoperdaceae*: *Abstoma*, *Apioperdon*, *Bovista*, *Bovistella* Morgan, *Bryoperdon*, *Calbovista*, *Calvatia*, *Disciseda*, *Fuscospina* R.L. Zhao & J.X. Li, *Gastropila*, *Lycoperdon*, *Lycoperdia*, *Morganella*, *Pseudoperdon* R.L. Zhao & J.X. Li, *Sinoperdon* R.L. Zhao & J.X. Li, and *Tortoperdon*.

Morphologically, *Longipediperdon spineum* resembles *Lycoperdon pulcherrimum* in subglobose to obpyriform basidiomata and *Lycoperdon*-type capillitium. However, *Lycoperdon pulcherrimum* differs from *Longipediperdon spineum* by having larger basidiomata (20–50 mm in diameter × 15–50 mm in height vs. 8–15 mm in diameter × 9–14 mm in height) and globose basidiospores (Bates et al. 2009). A detailed morphological comparison between the new genus *Longipediperdon* and the other 17 genera is provided in Table 3.

**Table 3.** Morphological comparison between the new genus *Longipediperdon* and the other 17 genera in the family *Lycoperdaceae*.

**Species name**	**Basidiomata**	**Exoperidium**	**Endoperidium**	**Gleba**	**Capillitium**	**Paracapillitium**	**Basidiospores**	**References**
* Abstoma *	Subglobose, stoma absent	Fragile, breaking away irregularly	Fragile, dehisces by irregular rupture	Firm at maturity	*Calvatia*-type occasionally branched, smooth	—	Apedicellate, globose	Cunningham 1926
* Apioperdon *	Obovoid to obpyriform	—	—	—	*Lycoperdon*-type	—	Smooth to minutely ornamented	Kreisel 1962
* Arachnion *	Small-sized,	—	Disintegrates at maturity	Develops minute peridioles resembling sand grains	Absent or poorly developed	—	Exhibit reticulate ornamentation	Cortez et al. 2013
* Bovista *	—	Persistent spines	—		*Bovista*-type or *Bovista*-*Lycoperdon* type	—	Ellipsoid, pedicellate	Li et al. 2024
* Bovistella *	Medium-sized, distinct pseudo-diaphragm	—	—	—	Fragile, and walls with abundant pits of irregular outline	—	Subglobose to slightly ellipsoid	Li et al. 2024
* Bryoperdon *	Small, ovoid, with mycelial cords	—	—	—	*Lycoperdon* -type	—	Smooth to minutely pustulose-verrucose	Vizzini and Ercole 2017
* Calbovista *	Medium to large, top-shaped	Coriaceous, fall away from top downward at maturity	—	Fragile, dark umber at maturit	Abundant discrete, ochraceous yellow, antler-like	—	—	Seidl 1995
* Calvatia *	Globose to pyriform, turbinate	Dehiscence of peridium occurring by irregular fragmentation	—	Pulverulent to cottony	*Calvatia*-type	—	Smooth to verrucose and echinate	Li et al. 2024
* Disciseda *	Globose to globose-depressed	Covered by a sand case	—	—	*Calvatia*-type, commonly wavy	—	Smooth to verrucose, and shortly pedicellate	Li et al. 2024
* Fuscospina *	Lycoperdoid, dark brown appearance	Adorned with conical and curved warts	Essile, 6–20 mm in diameter, obvious oral margin ring	—	*Lycoperdon*-*type capillitium with pits*	—	Covered with rounded warts	Li et al. 2024
* Gastropila *	Almost globose, dehiscing in an irregularly radiate-stellate manner	Fragile	Corky-spongy	—	Smooth threads, sparsely branched, not easily broken, much entangled	—	Smooth	Homrich and Wright 1973
* Longipediperdon *	Subglobose to obpyriform	With densely arranged spines structures	Papery and membranous	Cottony when young, pulverulent when mature	*Lycoperdon*-type, occasionally branched, thick-walled, no septa	Abundant	Ovoid, and oblong, with short spines, with a long pedicel	Present study
* Lycoperdon *		Echinate	Smooth	—	—	—	Globose to subglobose, usually ornamented with minute warts	Li et al. 2024
* Lycoperdia *	Pyriform, and subglobose to globose when dry	Densely arranged tomentose structures	Papery and fragile	Cottony texture when dry	*Lycoperdon*-type, branched, thick-walled, no septa	Composed of chains of colorless inflated cells, branched, no septa	Globose to subglobose, with distinct spines, with a short pedicel	Yang et al. 2025c
* Morganella *	Depressed, globose to pyriform	—	—	Consists of abundant paracapillitium	Absent	Abundant	Asperulate to echinulate	Li et al. 2024
* Pseudoperdon *	Globose to subglobose	—	—		*Lycoperdon*-type, with abundant large irregular pores	—	—	Li et al. 2024
* Sinoperdon *	Lycoperdoid	Echinate exoperidium covered with conical spines	—	—	*Lycoperdon*-type, thick walls	Abundant	Verrucose, long pedicels	Li et al. 2024
* Tortoperdon *	More or less subglobose to tuberiform	Present as a thin and easily removable layer of floccose to furfuraceous squamules	Fragile	Cottony	*Lycoperdon*-type to *Bovista*-*Lycoperdon* type, thick walls	—	Subglobose to broadly ellipsoid, not or shortly pedicellate	Yang et al. 2025b

As pivotal drivers of global nutrient cycling, fungi possess immense industrial and ecological potential (Merényi et al. 2023; Niego et al. 2023; Dong et al. 2025b). In terrestrial ecosystems, their reliance on plant biomass as a carbon source has driven co-evolution, producing diverse lifestyles such as parasitism, mutualism, and saprotrophy (Wijesinghe et al. 2025). *Lycoperdaceae* is a well-studied family within *Basidiomycota* (Larsson and Jeppson 2008; Krakhmalnyi et al. 2023). However, the present study reports a new genus and a new species from China, demonstrating that considerable undescribed diversity still exists within this group. Given the ecological importance and potential applications of this family, future work should prioritize comprehensive sampling and assessment to better elucidate their global distribution patterns and evolutionary history.

## Supplementary Material

XML Treatment for Longipediperdon

XML Treatment for Longipediperdon spineum

## Additional information

### Artificial Intelligence (AI) use

The authors accept full responsibility for the content of the manuscript, including the disclosure of any use of AI.

No AI tools were used in the preparation of this manuscript.

### Author ORCIDs

X. Yang https://orcid.org/0009-0009-7755-0581

S. Duan https://orcid.org/0009-0005-3122-0318

S. Li https://orcid.org/0009-0009-0396-3244

X. Gao https://orcid.org/0009-0002-0836-9315

R. Yang https://orcid.org/0009-0003-4574-9277

D. Jiang https://orcid.org/0009-0006-3441-9119

H. Zhou https://orcid.org/0000-0002-0724-5815

C. Zhao https://orcid.org/0000-0002-8668-1075

S. Shen https://orcid.org/0000-0002-7114-092X

### Data availability

All of the data that support the findings of this study are available in the main text.
